# Effects of Glycerol Monooleate on Improving Quality Characteristics and Baking Performance of Frozen Dough Breads

**DOI:** 10.3390/foods14020326

**Published:** 2025-01-20

**Authors:** Haocheng Liu, Jiguo Yang, Yujuan Xu, Jing Wen, Jinfeng Zhou, Zhijie Xu, Jian Li, Xueke Sun, Weili Si

**Affiliations:** 1 Sericultural & Argi-Food Research Institute, Guangdong Academy of Agricultural Sciences/Key Laboratory of Functional Foods, Ministry of Agriculture and Rural Affairs/Guangdong Key Laboratory of Agricultural Products Processing, No. 133 Yiheng Street, Dongguanzhuang Road, Tianhe District, Guangzhou 510610, China; ansishc@163.com (H.L.); guoshuxuyujuan@163.com (Y.X.); jingw988@163.com (J.W.); zhjiexu@163.com (Z.X.); 2 School of Food Science and Engineering, South China University of Technology, Guangzhou 510641, China; yangjg@scut.edu.cn (J.Y.); zhoujinfengau@outlook.com (J.Z.); 3 Heyuan Branch, Guangdong Laboratory for Lingnan Modern Agriculture, Heyuan 517000, China; 4 Dongguan Hsu Fu Chi Food Co., Ltd., Dongguan 523118, China; lj8029@163.com

**Keywords:** loaf, emulsifier, water distribution, microstructure, bakery product quality

## Abstract

This study investigated the impact of glycerol monooleate (MO) at varying levels (0.3%, 0.6%, 0.9%, and 1.2%) on the quality and baking properties of frozen dough. Low-field NMR and MRI were used to analyze the moisture distribution, water migration, and structural changes during frozen storage. The results indicated that MO reduced the content of free water, leading to a decrease in the spin–spin relaxation time of free water (T23). At the same time, the increase in the content of bound water resulted in an increase in the spin–spin relaxation time of bound water (T21). Rheological and SEM analyses revealed that MO preserved the dough’s microstructure and improved its rheological properties, reducing mechanical damage and inhibiting free water crystallization. This study found that by 8 weeks of frozen storage, the frozen dough containing 0.6% MO exhibited the best fermentation performance, with a larger fermentation volume and specific volume, and lower bread hardness, measuring 80 mL, 3.48 mL/g, and 1.10 N, respectively. These findings highlight MO’s potential in terms of enhancing frozen dough quality by maintaining the moisture balance and structural integrity during storage, offering a practical approach to improving bakery product quality.

## 1. Introduction

The baking industry widely uses frozen dough technology, and the consumption of baking products (such as croissants, Chinese steamed bread and pizza) made from frozen dough has increased due to its convenient characteristics. However, water inside the frozen dough freezes and expands to form ice crystals during freezing storage, which causes disruption to the gluten network and loss of yeast viability [1]. They are regarded as the primary reasons for the quality worsening of frozen dough [2,3]. Furthermore, its quality deteriorates during freezing storage, leading to a drop in the fermentative volume of dough, lower specific bread volume and hardened bread crumb texture. Therefore, for obtaining soft and delicious bread, it is necessary for formation and growth control of ice crystals. In a previous study, the emulsifiers used as additives received most attention, including sugar ester and sodium stearoyl lactylate [4], glycerol [5], calcium stearyl lactylate (CSL) and polysorbate 80 [6], diacetyl tartaric acid ester of mono(di)glycerides (DATEM) [7], etc. These emulsifiers delayed moisture migration between gluten and starch, which stabilized the gluten network [8]. Additionally, they might promote aggregation of the gluten networks in dough by binding to the protein hydrophobic surfaces [9]. Emulsifiers, including DATEM and CSL, formed presumably hydrogen bonds with glutamine [10]. Firstly, emulsifiers interact with gluten proteins, stabilizing the gluten network by aligning the hydrophobic regions, which enhances the dough strength and elasticity. Secondly, emulsifiers form complexes with amylose, delaying starch retrogradation during frozen storage, which helps to maintain dough softness and elasticity. Thirdly, by improving the water distribution within the dough, emulsifiers reduce the formation of large ice crystals, thereby protecting the cellular structure and minimizing damage during freezing and thawing. The mechanisms collectively contribute to improving the freeze–thaw stability of the dough, retaining the gas-holding capacity, and ultimately enhancing the quality of the baked products. These complexes improve the stability of the protein network, contributing to the development of texture and specific volume. Mono- and diglycerides of fatty acids (MDGs) have been widely used as emulsifiers and account for approximately one-third of the emulsifiers employed in the baking industry [11]. Commercial MDGs are normally formed by the reaction of glycerol with stearic acid, which is the most common fatty acid [12]. However, their performance when prepared from other fatty acids (including palmitic acid, linoleic acid, oleic acid, behenic acid, lauric acid and linolenic acid) is less well documented in the study of frozen dough. Glycerol monooleate (MO) consists of one molecule of oleic acid and one molecule of glycerol, and it is classified as one of the mono- and diglycerides of fatty acids. It has been derived from glycerol and oleic acid and is widely utilized in various industries, including food, pharmaceuticals, and cosmetics, owing to its emulsifying, stabilizing, and surfactant properties. MO has been extensively studied for its ability to form stable emulsions, improve food texture, and enhance the bioavailability of several nutrients and active compounds. Therefore, the purpose of this research is to evaluate the effects of various MO levels on frozen dough in terms of the water distribution, rheological properties, microstructure, fermentative volume and bread quality. After different MO levels are added to the frozen dough, LF-NMR is adopted to systematically detect the moisture distribution and free water (freezable water) content, and dynamic rheological measurement and SEM are used to measure the microstructure and viscoelastic properties. In addition, the quality changes are further determined by fermentation volume and baking. The results should provide new insights into the use of a new kind of emulsifier for MO.

## 2. Materials and Methods

### 2.1. Materials and Reagents

The wheat flour (13.70% protein, 11.98% moisture, 25.00 kg/bag) for commercial breadmaking was purchased from Lam Soon Hong Kong Group Co., Ltd., Hongkong, China.; the shortening (20.00 kg/bag) was purchased from Namchow Group, China; the instant dry yeast (0.50 kg/bag) was purchased from Angel Yeast Co., Ltd., Shanghai, China; the glycerol monooleate was purchased from Shandong Binzhou GIN&ING New Material Technology Co., Ltd., Binzhou, China; and the salt and sugar were purchased from a market in Nanchang. All the solutions and emulsions were prepared using double-distilled water (Milli-Q; Millipore, Bedford, MA, USA). Unless otherwise specified, all the other chemicals used were of analytical grade.

### 2.2. Frozen Dough Preparation and Treatment

The frozen dough recipe consisted of wheat flour (55.00 g), water (28.00 g), sugar (10.00 g), salt (0.50 g), instant dry yeast (0.50 g), shortening (6.00 g), and glycerol monooleate (0.30, 0.60, 0.90 or 1.20 g/100 g, based on the weight of wheat flour, *w*/*w*). The frozen dough without glycerol monooleate was considered to be the control group.

After mixing all the ingredients at a low speed for 30 min in a dough mixer (B20G, Guangdong, China), the frozen dough was molded. Then, by hand-shaping into rounds, it was separated into 30 g pieces to wrap in polyvinyl chloride films, pack with polyethylene bags, and reserve at −18 °C for 0 week, 2 weeks, 4 weeks, 6 weeks and 8 weeks.

### 2.3. Bread Preparation

The 30 g dough was thawed at 4 °C for 2 h [13], before the polyvinyl chloride films were dismantled. Then, it was placed into a cylindrical stainless-steel mold with a diameter of 60 mm and a height of 50 mm, and fermented at 37 °C and 80% (RH, relative humidity) for 2 h. After the fermentation was completed, the dough was placed into a preheated oven set at 200 °C and baked for 8 min. Finally, the baked bread was allowed to cool for 60 min at room temperature and atmospheric pressure before being used for testing.

### 2.4. LF-NMR Measurement

To estimate the moisture distribution of the frozen dough samples, LF-NMR (MicroMR-25, Niumag Electric Corporation of Shanghai, China) was utilized at an operating resonance frequency of 21.986 MHz. The sample (3.00 g), which was contained in a diameter glass tube (20 mm), was thawed at room temperature for 1 h [14]. After thawing, the glass tube containing the frozen dough sample was transferred into the LF-NMR probe. The moisture distribution was assessed by measuring the spin–spin relaxation time (T2) using a Carr–Purcell–Meiboom–Gill (CPMG) pulse sequence. The following parameters were used for the NMR measurement: the length of the 90 pulse was (P90) = 7 μs; the length of the 180 pulse was (P180) = 14 μs, TD = 156,992, NECH = 5000, SW = 100 kHz, RFD = 0.80 ms, TW = 1500 ms, RG1 = 10 db, DRG1 = 3, and NS = 64; and the temperature of the magnet was 32 °C.

The T2 fitting procedure fitted the CPMG data, and the corresponding water contents were obtained from the cumulative area under each peak. The proton image of the moisture distribution in the dough samples was obtained via magnetic resonance imaging (MRI) by LF-NMR, and the pseudo-color image of the moisture distribution in the corresponding dough was acquired by Matrix Laboratory (MATLAB). In the pseudo-color images, an increase in water molecules in the dough tissue was indicated by changing from blue to bright yellow to red.

### 2.5. Dynamic Rheological Measurement

After being thawed at 4 °C for 2 h, the dynamic rheological properties of the samples were evaluated at 25 °C by a Discovery HR-1 hybrid rheometer (TA Instruments, New Castle, DE, USA).

The samples were then placed between parallel plates (40 mm diameter and 2 mm gap) and the excess was carefully trimmed. To avoid their moisture evaporation during the tests, their edges were coated with a thin layer of silicone oil. Before each oscillatory measurement, they sat for 5 min to relax the residual stress. Strain sweeps were performed at a constant frequency (1 Hz) from 0.01% to 100.00% for determining the linear viscoelastic range (LVR) of the samples. Afterward, oscillatory frequency sweep measures were performed over 0.1~10 Hz of the scanning frequency with 0.1% of constant strain and 5 points per decade to obtain the loss modulus (G″), storage modulus (G′), and loss tangent (tan δ = G″/G′).

### 2.6. Dough Analysis by SEM

After the dough was prepared and subjected to freezing and frozen storage, samples were taken for freeze-drying to examine the microstructure. The samples that were frozen at −80 °C for different times (0 and 8 weeks) were freeze-dried. After freeze-drying, the dough samples were cut into small pieces and mounted onto aluminum stubs using double-sided adhesive tape. The samples were then coated with a thin layer of gold to enhance the conductivity and prevent charging during the SEM analysis. The samples were then observed and images were captured using a QUANTA 250 FEI SEM (Hillsboro, OR, USA) at a magnification of 3000× with an accelerating voltage of 15 kV.

### 2.7. Fermentation Volume of Frozen Dough with Different MO Levels

The fermentative volumes of the frozen dough samples were measured according to a method adopted from [15]. The 30 g dough was thawed at 4 °C for 30 min, before the polyvinyl chloride films were dismantled. The thawed dough samples (30 g) were placed in sterilized graduated cylinders (250 mL) and fermented in the prover at 37 °C and 80% (RH) for 2 h. The fermentative volume (mL) of the dough samples was recorded.

### 2.8. Evaluation of Bread Quality

After being baked, the breads were cooled down at room temperature for 1 h. Their specific volumes were then calculated by the rapeseed displacement method according to AACC Approved Method 10-05 [16]. The displacement of rapeseeds caused by the bread was measured, and the specific volume was calculated by dividing the volume of displaced rapeseeds by the weight of the bread.

Texture profile analysis (TPA) was adopted to measure the hardness of the bread crumbs using the texture analyzer TA. XT. plus (Stable Micro Systems Ltd., Surrey, UK). The analysis was conducted with slight modifications to the standard procedure. Each bread loaf was sliced into approximately 1.5 cm thick pieces to ensure uniform testing. The aluminum cylindrical probe P/36R was used with the test parameters set as 1 mm/s pre-test speed, 5 mm/s test speed, 5 mm/s post-test speed, 50% compressive strain, and 5 g trigger force.

### 2.9. Statistical Analysis

All the experiments were carried out three times, from which the data were presented as the mean ± standard deviation and compiled in Origin 2019. Afterward, the one-way ANOVA and Tukey’s multiple comparisons were adopted for significance analysis in the software IBM SPSS Statistics 23 (SPSS Inc., Chicago, IL, USA), and *p* < 0.05 would indicate a statistically significant difference.

## 3. Results and Discussion

### 3.1. Effect of MO on Water Migration and Moisture Distribution of Frozen Dough

The study of this frozen dough looked like this: as is known to all, the water content and its distribution play a crucial role in the organization of baked goods (e.g., bread, cakes, biscuits, etc.). Therefore, it is crucial to understand the quantity, physical state and location of water for the storage, defrosting and baking of frozen dough [17].

**Figure 1:**
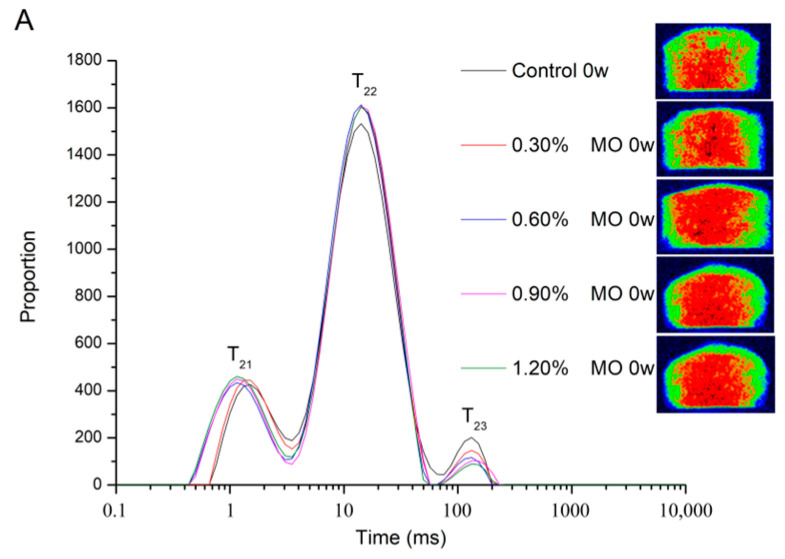

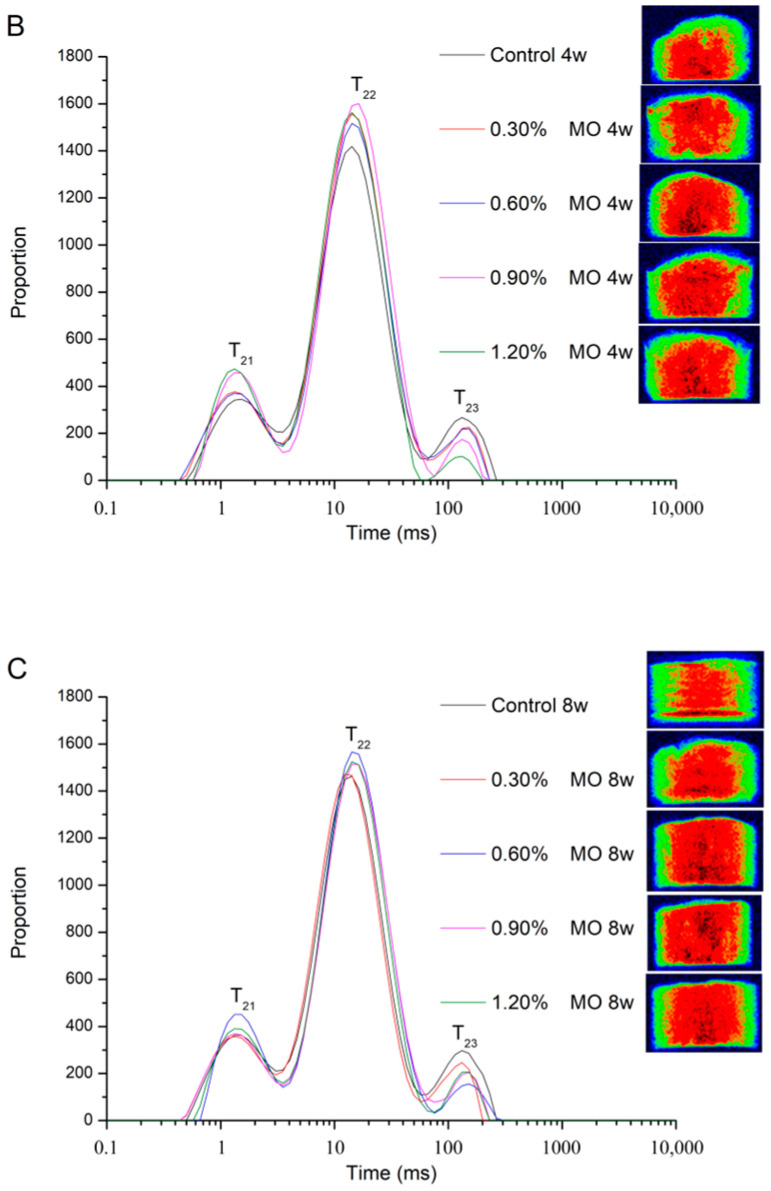
T2 relaxation time distribution curves and pseudo-color images of the dough samples with different contents of glycerol monooleate (MO). (**A**) Frozen storage at 0 week (fresh dough); (**B**) frozen storage at 4 weeks; and (**C**) frozen storage at 8 weeks.

In this research, the molecular mobility of the water and the relaxation time distribution of the dough were studied by the spin–spin relaxation time (T2). The typical distribution curve is presented in Figure 1, which includes three proton populations: T21 (0.01–3.05 ms), T22 (3.05–75 ms) and T23 (75–500 ms), representing bound water, fixed water and free water, respectively [10,18], and the related peak area ratio corresponds to the water quantity. The moisture distribution in the control group was more variable than in the fresh dough with MO at 0 week. The area ratio of T21 of the dough improved from 17.17% to 19.47%, whereas T23 decreased from 3.67% to 1.87% with the increase in the MO addition level. The area ratio of T21 and T22 increased in the dough with MO more than that in the control group, and the area ratio of T23 decreased. The proportion of bound water and free water was negatively correlated with the amount of MO addition, while the proportion of fixed water was positively correlated with it. Therefore, this difference demonstrated that MO was able to bind more water molecules to form bound water, which was consistent with its hydrophilic structure. After 8 weeks of freezing storage, the T21 of the control group declined from 17.33% to 15.30%, and the T22 decreased from 78.23% to 75.70% too, but the T23 increased from 4.43% to 9.00%. The increase in free water, as the decrease in bound water and immobilized water, might be attributed to the breakage of gluten. This tendency was attributed to the mechanical damage induced by ice crystals during frozen storage [19,20]. In contrast, when the frozen dough was supplemented with MO, it slowed the rise of free water and the decline of bound water and fixed water progressively. As mentioned above, the MO could reduce the impact of freezing storage on the deterioration of frozen dough and play a positive role in it. Magnetic resonance imaging (MRI) has lately been deemed a fast, direct, and non-destructive method for food analysis [21]. In Figure 1, the differences in the various frozen storage times of the dough are presented by pseudo-color images. The more water was in the frozen dough, the higher the proton density and the darker the image. The color images of the fresh dough were well distributed, with clear edges, indicating an even moisture distribution from surface to interior. But after frozen storage, this state was broken. Specifically, after 8 weeks of frozen storage, there were more red or brown areas in the dough, more blue or yellow areas on the surface, and more red or brown areas in the internal area, which possibly resulted from the water migration during frozen storage. It can be hypothesized that the water loss mainly happened on the dough surface due to frozen storage. After adding MO to the dough, the red or brown area was more pronounced than in the control group, which means that the MO-added dough retained more moisture during frozen storage. Therefore, the addition of MO can improve the water migration, thereby delaying the water loss.

### 3.2. Effect of MO on the Dynamic Frequency Sweep Measurements of Frozen Dough

**Figure 2:**
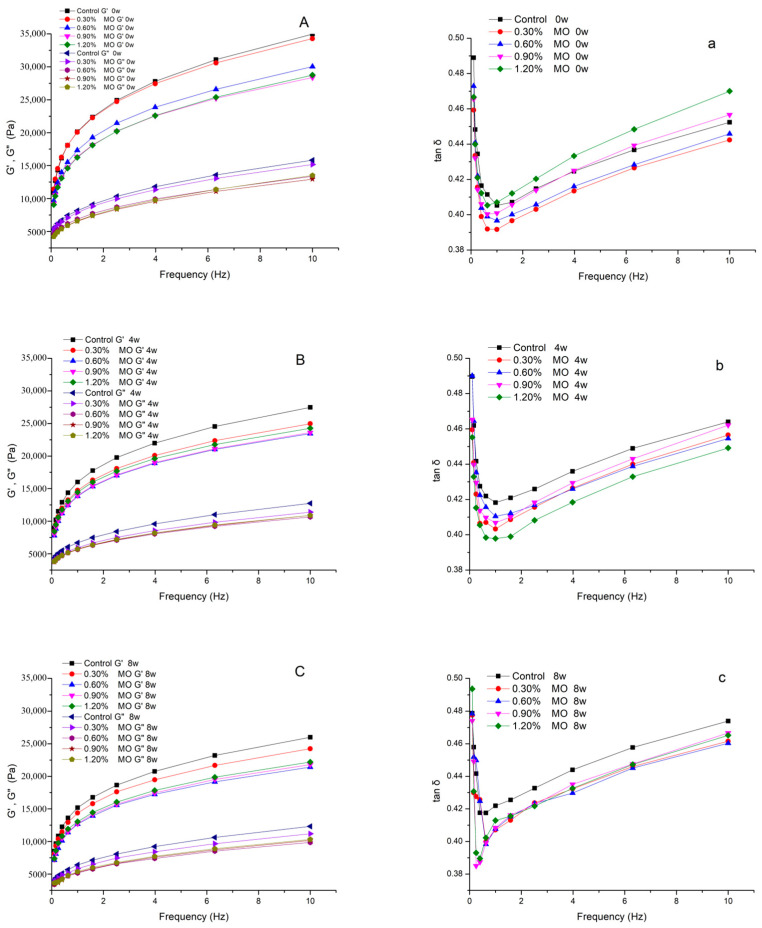
Viscoelastic behavior of different MO level groups of frozen dough at 0 week, 4 weeks, and 8 weeks. Note: G′, storage modulus; G″, loss modulus; tan δ, loss tangent; MO, glycerol monooleate; 0 w, frozen storage 0 week; 4 w, frozen storage 4 weeks; 8 w, frozen storage 8 weeks. (**A**) Storage modulus and loss modulus of frozen dough in frozen storage at 0 week; (**a**) loss tangent of frozen dough in frozen storage at 0 week; (**B**) storage modulus and loss modulus of frozen dough in frozen storage at 4 weeks; (**b**) loss tangent of frozen dough in frozen storage at 4 weeks; (**C**) storage modulus and loss modulus of frozen dough in frozen storage at 8 weeks; and (**c**) loss tangent of frozen dough in frozen storage at 8 weeks.

The wheat dough is a viscoelastic material with the properties of viscous liquids and elastic solids [22], and its elasticity and viscosity are reflected in the storage modulus (G′) and loss modulus (G″), respectively. The results showed that the frozen treatment improved the rheological properties. Figure 2 shows the influence of MO on the rheological parameters of the dough at 0, 4, and 8 weeks. The G′ values were higher than the G″, so the tan δ of all the doughs was less than 1, indicating the greater elasticity of the dough. The presence of increasing amounts of MO in the dough resulted in a reduction in both G′ and G″, indicating that MO addition resulted in softening behavior in terms of the dough. This trend was observed with similar viscoelastic behavior of dough when mixed with modified whey protein [23,24]. After the MO and the control group were added to the frozen dough, the G′ and G” decreased with the freezing time. This phenomenon occurred because of the water crystallization and ice recrystallization in the dough. The water in the dough would crystallize during the frozen period, and the sizes of the ice crystals might grow larger due to the water redistribution with the increasing frozen storage times, leading to mechanical damage to its gluten network. A similar trend was previously reported for frozen dough [10,25,26]. As the frozen storage times increased, the tan δ rose, and that of the control group was higher than that of the MO group. It was observed that the MO could retard the increase in the tan δ, meaning that the addition of MO could mitigate the deterioration.

### 3.3. Effect of MO on the Microstructure of Frozen Dough

**Figure 3:**
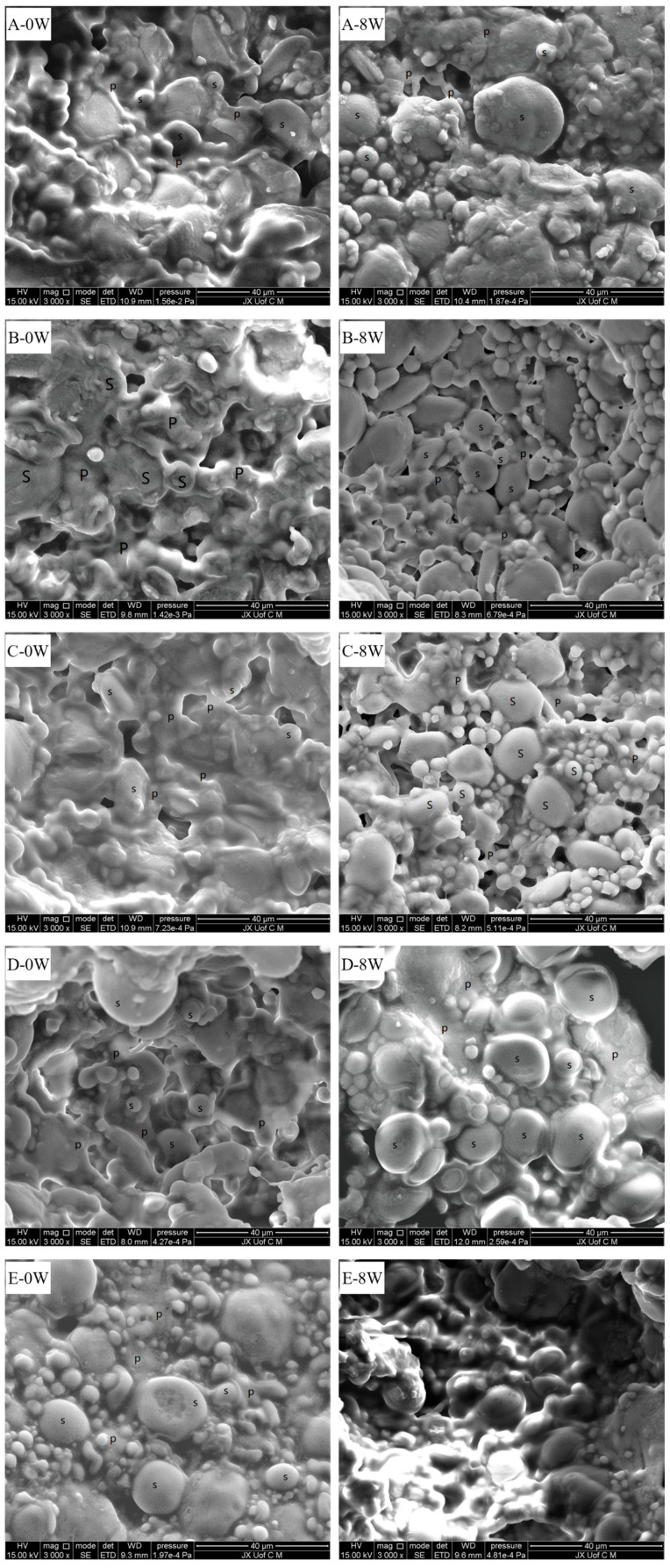
Scanning electron microscope (SEM) images of the dough samples after the same frozen storage time: (**A**) the control groups; (**B**) 0.3% MO; (**C**) 0.6% MO; (**D**) 0.9% MO; and (**E**) 1.2% MO. P: gluten network. S: starch granules. Note: 0w, frozen storage 0 week; 8w, frozen storage 8 weeks.

SEM was used to observe the frozen dough’s microstructure. During dough preparation, a continuous membranous gluten network was formed by the interaction between glutenin and gliadin through water, in which the starch granules were wrapped [6,27]. Its strength and stability were important to the end-product’s quality (e.g., bread), such as the specific volume and texture of the bread. In Figure 3 can be observed the micrographs corresponding to the control and the dough containing different MO levels before and after 8 weeks of frozen storage. It can be seen from Figure 3(B-0w, C-0w, D-0w and E-0w) that the microstructure of the dough was smoother and denser, and the wrapped small starch granules in the continuous gluten network were better than the control (Figure 3(A-0w)). In Figure 3(A-8w), after 8 weeks of freezing storage, the starch granules were separated from the gluten network, indicating that the binding of the latter to the former was disrupted. In addition, the latter was poor in its continuity, and large gas voids were present. These results were similar to the study in [28]. Also, the microstructure of the control (Figure 3(A-8w)) indicated that the shapes of small starch granules were unchanged but the original smooth surfaces of large granules become rough during frozen storage [29]. All these changes resulted from the formation and growth of ice crystals in the dough [21,30]. With the addition of various MO levels, the starch granules separated from the gluten network situation became less notable, as shown in Figure 3(B-8w, C-8w, D-8w and E-8w)). The different MO levels improved the frozen dough to a varying extent. The frozen dough with high MO levels had a smooth, thick and continuous gluten network. The reason for this was probably that the addition of MO can change the growth of the ice crystals and inhibit large ice crystals being formed, thus retarding the deterioration of the gluten network.

### 3.4. Effect of MO on the Fermentative Volume of Frozen Dough

**Figure 4:**
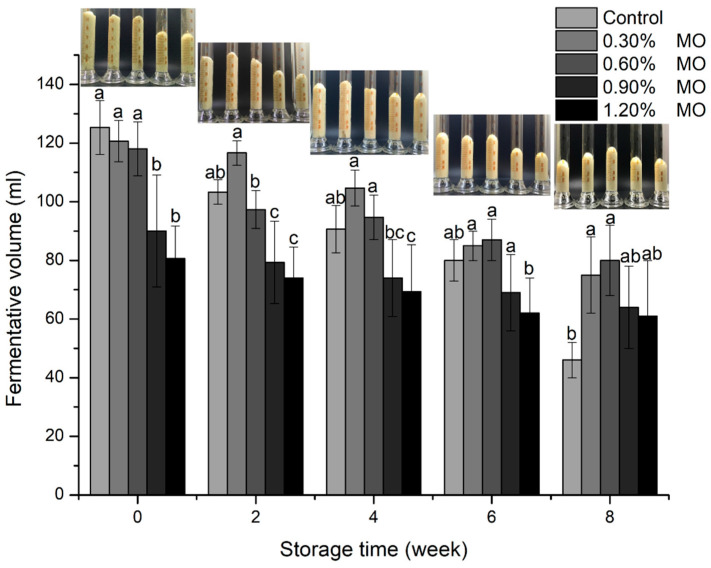
Fermentative volume of different MO level groups of frozen dough after the same frozen storage time. Different letters indicate a significant difference between the values (*p* < 0.05).

The fermentative volume was of great importance in terms of the bread texture. The fermentative volume of dough is related not only to the gas (CO_2_) productivity of the yeast but also to the capacity to hold the gas produced during the development stage. The gas retention ability was largely determined by the viscoelastic gluten network integrity [31,32], and thus the fermentative volume was used to determine the gluten network stability and strength during the fermentation of frozen dough. As shown in Figure 4, a significant reduction was observed in the fermentative volume of the dough with the addition of higher MO levels at 0 week. This trend might be attributed to the competition for water by the higher MO leading to insufficient hydration of gluten proteins. In the 8 weeks of frozen storage, the fermentative volume of the control group and the different MO content groups of the frozen dough showed a decreasing trend. The control group exhibited the greatest decrease in the fermentative volume, with a reduction of 63.30%. The fermentative volumes of the dough with different MO levels decreased by 37.85%, 32.20%, 28.89%, and 24.38%, respectively. Therefore, the addition of MO could protect the internal structure of the dough to some extent and reduce the disruption of the gluten network by frozen storage.

### 3.5. Effect of MO on Bread Quality

**Figure 5:**
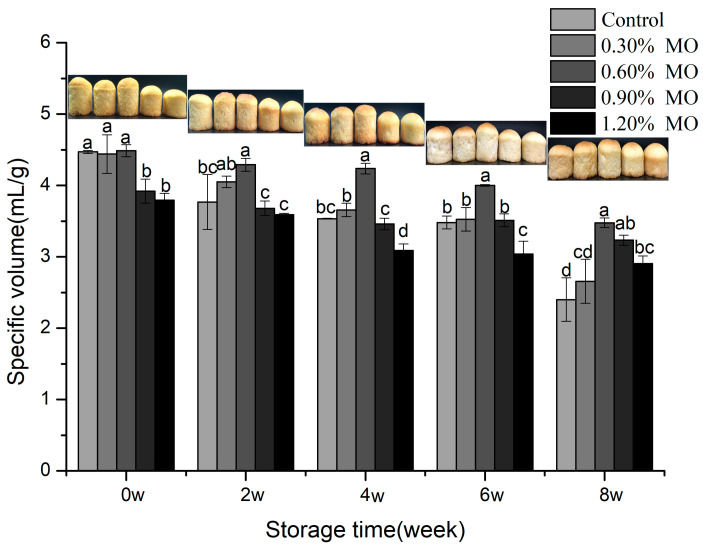
Specific volume of the different MO breads after the same frozen storage time. Different letters indicate a significant difference between the values (*p* < 0.05).

**Figure 6:**
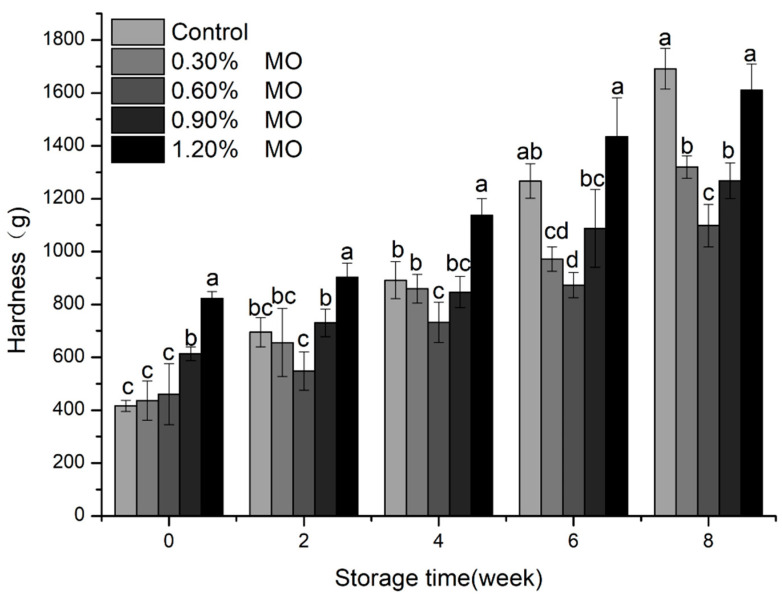
Hardness of different MO level groups of frozen dough after the same frozen storage time. Different letters indicate a the significant difference between the values (*p* < 0.05).

The bread quality was evaluated in terms of the specific volume and hardness. The influence of different MO levels on the specific volume of bread is presented in Figure 5. A descending tendency concerning the specific volume of bread contributed to by an increase in the MO levels was observed at 0 week. The reason might be attributed to the competition for water by the higher MO, leading to insufficient hydration of gluten proteins and starch during baking. The bread in specific volume declined with prolonged frozen storage, which resulted from the breaking influence of ice crystals on the gluten network. In these breads, a smaller specific volume reduction in the higher MO levels was observed. This finding could indicate that the addition of MO, as an emulsifier, caused more water molecules to bind, which reduced the disruption to the gluten network by ice crystals. These structural differences might have an impact on other properties of quality, such as the textural characteristic: hardness. As shown in Figure 6, the change in the bread hardness was not significant in the lower MO groups and the control (*p* < 0.05) at 0 week, while the higher MO groups changed more significantly (*p* < 0.05). This was probably attributed to the emulsifier (such as sodium stearyl lactate) interacting with the gluten network, increasing the gluten network polymerization and resulting in higher hardness of the bread [33,34]. The hardness of all the bread rose with the freezing time, but at the same freezing time, the hardness of the bread with lower MO groups was the smallest. Emulsifiers not only interact with gluten proteins but also form complexes with starch, particularly amylose, which helps to delay retrogradation and maintain dough softness during frozen storage.

## 4. Conclusions

After studying the effects of various levels of MO on the moisture distribution, rheological properties, microstructure, fermentative volume and bread quality of frozen dough in this paper, LF-NMR and MRI tests revealed that the addition of MO restricted the moisture distribution and slowed the increase of T23 (free water) during frozen storage. Moreover, with the increase from 0.30% to 1.20%, T21 (bound water) rose and T23 decreased gradually. It can be concluded that MO could weaken the effect of frozen storage on water migration and affect the moisture distribution in the dough. However, MO maintained the continuous gluten structure during frozen storage, suggesting inhibitory mechanical damage to the free water crystallization. As for the rheological properties and microstructure, rheology tests and SEM showed that various MO levels mitigated their deterioration in frozen storage. Based on the study of the frozen dough, the sample with 0.6% MO demonstrated the most favorable results across the examined quality parameters. Therefore, the addition of a suitable level of MO resulted in a largish fermentative volume of the frozen dough and a higher specific volume and lower hardness of the bread.

## Data Availability

The original contributions presented in this study are included in the article/Supplementary Materials; further inquiries can be directed to the corresponding author.

## Supplementary Materials

The following supporting information can be downloaded at https://www.mdpi.com/article/10.3390/foods14020326/s1, Table S1 Correlation of MO Content with Dough Water Content; Table S2: Moisture Distribution in Frozen Dough with MO; Table S3: T2 Relaxation Time Image of Dough with MO; Table S4: Fermentative Volume of Frozen Dough with MO; Table S5: Specific Volume of Bread with MO; Table S6: Peak Areas of Water States in Frozen Dough with MO and Storage Time.
