# Supplementary material for: Effects of Glycerol Monooleate on Improving Quality Characteristics and Baking Performance of Frozen Dough Breads
Source: Foods. 2025 Jan 20;14(2):326. doi: 10.3390/foods14020326 (PMC11765111; doi:10.3390/foods14020326)
Supplement: Supplementary file 1 [file foods-14-00326-s001.zip › Table S1.pdf]

Table S1 Correlation analysis of frozen dough with different content of MO on water content

Index		0 week	4 weeks	8 weeks
Fixed water	Pearson correlation analysis	0.019	0.796	0.603
	P -value	0.947	0.000	0.017
Bound water	Pearson correlation analysis	-0.375	-0.340	-0.327
	P -value	0.164	0.215	0.235
Free water	Pearson correlation analysis	0.019	-0.695	-0.637
	P -value	0.946	0.004	0.011
N		15	15	15

Note: N, sample size.
