# Supplementary material for: Effects of Glycerol Monooleate on Improving Quality Characteristics and Baking Performance of Frozen Dough Breads
Source: Foods. 2025 Jan 20;14(2):326. doi: 10.3390/foods14020326 (PMC11765111; doi:10.3390/foods14020326)
Supplement: Supplementary file 1 [file foods-14-00326-s001.zip › Table S2.pdf]

Table S2. The moisture distribution of T21, T22, and T23 in frozen dough with different concentrations of Glycerol Monooleate (MO)

The types of water	Sample	Frozen storage time (week)		
		0 week	4 weeks	8 weeks
T_{21} (%) Bound water	Control	17.33 ± 0.40c	16.23 ± 0.47c	15.30 ± 0.30c
	0.30% MO	17.17 ± 0.50c	16.63 ± 0.12c	16.00 ± 0.26b
	0.60% MO	18.27 ± 0.72b	17.50 ± 0.35b	16.73 ± 0.61a
	0.90% MO	18.67 ± 0.15ab	17.57 ± 0.21b	16.67 ± 0.06a
	1.20% MO	19.47 ± 0.42a	18.53 ± 0.29a	16.67 ± 0.21a
T_{22} (%) Immobilized water	Control	78.23 ± 0.81b	76.03 ± 0.91b	75.70 ± 1.18b
	0.30% MO	79.17 ± 0.47a	78.27 ± 0.80a	78.70 ± 1.40a
	0.60% MO	79.43 ± 0.31ab	77.97 ± 0.85a	78.10 ± 0.75a
	0.90% MO	78.97 ± 0.25ab	78.97 ± 0.25a	78.63 ± 0.80a
	1.20% MO	78.67 ± 0.42ab	78.90 ± 0.20a	78.53 ± 0.31a
T_{23} (%) Free water	Control	4.43 ± 0.40a	7.73 ± 0.81a	9.00 ± 0.89a
	0.30% MO	3.67 ± 0.57b	5.10 ± 0.70b	5.30 ± 1.28b
	0.60% MO	2.30 ± 0.44c	4.53 ± 1.19bc	5.17 ± 1.37b
	0.90% MO	2.37 ± 0.32c	3.47 ± 0.15cd	4.70 ± 0.80b
	1.20% MO	1.87 ± 0.23c	2.57 ± 0.35d	4.80 ± 0.10b
