# Supplementary material for: Effects of Glycerol Monooleate on Improving Quality Characteristics and Baking Performance of Frozen Dough Breads
Source: Foods. 2025 Jan 20;14(2):326. doi: 10.3390/foods14020326 (PMC11765111; doi:10.3390/foods14020326)
Supplement: Supplementary file 1 [file foods-14-00326-s001.zip › Table S3.pdf]

Table S3. T2 relaxation time pseudo-color image of the dough sample with different content of Glycerol Monooleate (MO).

Sample	0 week	4 weeks	8 weeks
Control	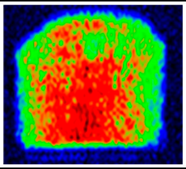	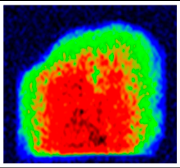	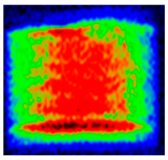
0.3% MO	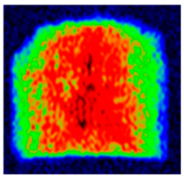	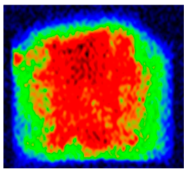	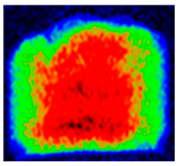
0.6% MO	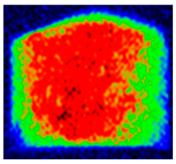	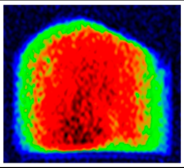	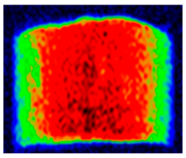
0.9% MO	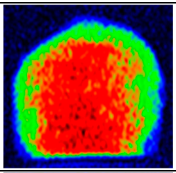	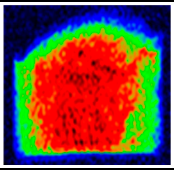	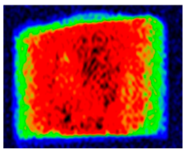
1.2% MO	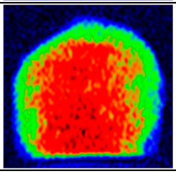	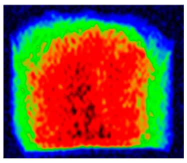	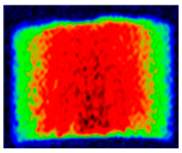
