# Supplementary material for: Effects of Glycerol Monooleate on Improving Quality Characteristics and Baking Performance of Frozen Dough Breads
Source: Foods. 2025 Jan 20;14(2):326. doi: 10.3390/foods14020326 (PMC11765111; doi:10.3390/foods14020326)
Supplement: Supplementary file 1 [file foods-14-00326-s001.zip › Table S4.pdf]

Table S4. Fermentative volume of different MO level groups of the frozen dough in the same frozen storage time.

Sample	0 week	2 weeks	4 weeks	6 weeks	8 weeks
Control	125.33±9.24a	103.33±4.16ab	90.67±8.08ab	80.00±7.00ab	46.00±6.00b
0.30% MO	120.67±7.02a	116.67±4.16a	104.67±6.11a	85.00±5.00a	75.00±13.00a
0.60% MO	118.00±9.17a	97.33±6.43b	94.67±7.57a	87.00±7.00a	80.00±12.00a
0.90% MO	90.00±19.08b	79.33±14.05c	74.00±13.11bc	69.00±13.00a	64.00±14.00ab
1.20% MO	80.67±11.02b	74.00±10.58c	69.33±16.04c	62.00±12.00b	61.00±19.00ab
