# Supplementary material for: Effects of Glycerol Monooleate on Improving Quality Characteristics and Baking Performance of Frozen Dough Breads
Source: Foods. 2025 Jan 20;14(2):326. doi: 10.3390/foods14020326 (PMC11765111; doi:10.3390/foods14020326)
Supplement: Supplementary file 1 [file foods-14-00326-s001.zip › Table S5.pdf]

Table S5. Specific volume of the different MO breads in the same frozen storage time.

Sample	0 week	2 weeks	4 weeks	6 weeks	8 weeks
Control	4.47±0.02a	3.77±0.39bc	3.53±0.01bc	3.48±0.09b	2.40±0.31d
0.30% MO	4.44±0.27a	4.05±0.08ab	3.66±0.10b	3.52±0.17b	2.66±0.31cd
0.60% MO	4.49±0.09a	4.29±0.09a	4.24±0.08a	4.00±0.01a	3.48±0.07a
0.90% MO	3.92±0.17b	3.68±0.10c	3.46±0.08c	3.51±0.09b	3.23±0.07ab
1.20% MO	3.79±0.10b	3.59±0.02c	3.09±0.10d	3.04±0.18c	2.90±0.11bc
