# Supplementary material for: Effects of Glycerol Monooleate on Improving Quality Characteristics and Baking Performance of Frozen Dough Breads
Source: Foods. 2025 Jan 20;14(2):326. doi: 10.3390/foods14020326 (PMC11765111; doi:10.3390/foods14020326)
Supplement: Supplementary file 1 [file foods-14-00326-s001.zip › Table S6.pdf]

Table S6. The raw data on the effects of different frozen storage times and various concentrations of Glycerol Monooleate (MO) on the peak areas of three physical states of water in frozen dough

Sample	Different states of water	Peak Area of 0 week	Peak Area of 4 weeks	Peak Area of 8 weeks
Control Group A	Bound water	3599.84	3252.34	3237.19
	Immobilized water	16594.86	15906.99	16648.87
	Free water	899.85	1571.37	1734.45
	Total	21094.55	20730.71	21620.51
Control Group B	Bound water	3639.89	3378.43	3172.57
	Immobilized water	16717.36	15443.27	15612.32
	Free water	897.24	1767.46	1919.64
	Total	21254.48	20589.16	20704.52
Control Group C	Bound water	3827.06	3435.07	3382.14
	Immobilized water	16623.51	15893.23	16157.45
	Free water	1045.45	1464.09	2102.89
	Total	21496.03	20792.38	21642.48
0.30% MO A	Bound water	3611.90	3589.77	3472.40
	Immobilized water	16853.22	17245.37	17241.79
	Free water	685.31	952.38	918.13
	Total	21150.42	21787.52	21632.32
0.30% MO B	Bound water	3559.07	3597.47	3381.12
	Immobilized water	16794.59	16901.54	17061.35
	Free water	911.03	1111.70	1071.29
	Total	21264.69	21610.71	21513.75
0.30% MO C	Bound water	3784.08	3633.25	3377.59
	Immobilized water	16824.62	16879.46	16036.90
	Free water	742.93	1280.74	1396.71
	Total	21351.63	21793.45	20811.20
0.60% MO A	Bound water	4090.86	3732.75	3625.69
	Immobilized water	16950.65	16671.10	17046.14
	Free water	377.91	792.92	1176.28
	Total	21419.42	21196.77	21848.11
0.60% MO B	Bound water	3804.56	3766.28	3563.13
	Immobilized water	17117.07	16732.50	17120.97
	Free water	544.91	862.62	1429.58
	Total	21466.55	21361.40	22113.68
0.60% MO C	Bound water	3857.56	3652.31	3749.30
	Immobilized water	17143.25	16455.25	17077.30
	Free water	559.46	1266.43	804.85
	Total	21560.27	21373.98	21631.45

0.90% MO A	Bound water	4021.39	3775.02	3535.72
	Immobilized water	16954.27	17037.67	16877.38
	Free water	561.20	711.30	836.13
	Total	21536.86	21523.98	21249.23
0.90% MO B	Bound water	4006.86	3836.29	3544.24
	Immobilized water	16832.25	16977.98	16820.80
	Free water	426.19	770.41	1016.40
	Total	21265.29	21584.67	21381.44
0.90% MO C	Bound water	3974.48	3786.06	3508.16
	Immobilized water	16996.18	17165.92	16329.61
	Free water	541.18	785.15	1158.31
	Total	21511.85	21737.13	20996.08
1.20% MO A	Bound water	4062.08	3833.42	3456.57
	Immobilized water	16892.31	16640.69	16390.09
	Free water	418.54	623.67	999.12
	Total	21372.92	21097.78	20845.78
1.20% MO B	Bound water	4158.07	3921.91	3413.38
	Immobilized water	16734.97	16603.88	16340.43
	Free water	357.24	468.51	969.63
	Total	21250.28	20994.29	20723.44
1.20% MO C	Bound water	4272.84	3955.11	3640.11
	Immobilized water	16836.42	16685.81	16806.27
	Free water	423.07	562.91	1058.55
	Total	21532.34	21203.83	21504.93
